# A Threshold Value for the Time Delay to TB Diagnosis

**DOI:** 10.1371/journal.pone.0000757

**Published:** 2007-08-22

**Authors:** Pieter W. Uys, Robin M. Warren, Paul D. van Helden

**Affiliations:** Division of Molecular Biology and Human Genetics, MRC Center for Molecular and Cellular Biology, DST/NRF Centre of Excellence for Biomedical TB Research, Faculty of Health Sciences, Stellenbosch University, Cape Town, Western Cape, Republic of South Africa; Medical University of South Carolina, United States of America

## Abstract

**Background:**

In many communities where TB occurs at high incidence, the major force driving the epidemic is transmission. It is plausible that the typical long delay from the onset of infectious disease to diagnosis and commencement of treatment is almost certainly the major factor contributing to the high rate of transmission.

**Methodology/Principal Findings:**

This study is confined to communities which are epidemiologically relatively isolated and which have low HIV incidence. The consequences of delays to diagnosis are analyzed and the existence of a threshold delay value is demonstrated. It is shown that unless a sufficient number of cases are detected before this threshold, the epidemic will escalate. The method used for the analysis avoids the standard computer integration of systems of differential equations since the intention is to present a line of reasoning that reveals the essential dynamics of an epidemic in an intuitively clear way that is nevertheless quantitatively realistic.

**Conclusions/Significance:**

The analysis presented here shows that typical delays to diagnosis present a major obstacle to the control of a TB epidemic. Control can be achieved by optimizing the rapid identification of TB cases together with measures to increase the threshold value. A calculated and aggressive program is therefore necessary in order to bring about a reduction in the prevalence of TB in a community by decreasing the time to diagnosis in all its ramifications. Intervention strategies to increase the threshold value relative to the time to diagnosis and which thereby decrease disease incidence are discussed.

## Introduction

In many underdeveloped countries, health-care is challenged by the task of coping with high incidence endemic TB. Although individual TB cases are effectively diagnosed and treated, incidence continues to increase in many settings. A plausible reason for this is that there are prolonged delays from the onset of TB disease in individuals to the time of eventual diagnosis. During such delays, an active case may infect numerous susceptible people thereby contributing to the perpetuation or expansion of the epidemic. The period from disease onset to reporting to a health care facility may be as long as 50 to 90 days [1]–[3], or even up to 162 days (mean value), as reported in Tanzania [4]. In the context of the transmission of an infectious disease, this delay period would therefore seem to be highly significant and to warrant close investigation to determine the extent to which it may be a factor aggravating the severity of the epidemic. Thus, all possible factors involved in the process of disease transmission during such periods need to be carefully analyzed.

In the past, standard epidemiological theory typically has not taken into account variations in the degree of infectiousness of individuals over time and furthermore, it has assumed homogeneous and unlimited mixing. The shortcomings of this approach and the concept of the basic reproduction number, R_0_, have been investigated by Liljeros et al [5]. More recently, a ‘risk index’ to predict the infectiousness of tuberculosis patients has been investigated [6] and heterogeneous mixing is receiving attention [7], [8]. These factors may well play a significant role and in order to account for them, the process of disease transmission needs to be quantified in terms of factors concerning the relationship between time and degree of infectiousness together with the number of available susceptible contacts and the time from onset of infectious disease to diagnosis. The combination of these factors will yield the probability, as a function of time, of a TB transmission event originating from an infectious person.

An examination of these factors in combination shows that if infectious cases are timeously detected and treated, the incidence of TB disease cases can be reduced. The maximum time period from start of infectiousness to diagnosis and treatment that will permit this is defined to be the threshold time. If sufficient cases are detected before the threshold time, an epidemic can be brought under control. This means that to curb an epidemic, diagnoses need to be made earlier or the threshold time needs to be increased. A combination of both strategies may be employed with the result that the threshold time can indeed be made longer than the delay time to diagnosis, or even indefinitely long.

This study is specific to a particular community [9] which is epidemiologically relatively isolated and which has a low HIV incidence of about 2%. Initially we assume that there is no migration into or out of the community and that the birth rate is equal to the average mortality rate. These attributes permit a very simple analysis. The potential to relax these restrictions on the analysis is discussed and it will be shown that the simple analysis is still valid in more general settings.

## Methods

In order to determine whether an epidemic is expanding or not it is sufficient to ascertain whether the cohort of infected persons is increasing or decreasing. This can be achieved by examining the contribution to the cohort of infected persons made by active cases infecting susceptible individuals. The cohort will be expanding if this contribution exceeds the losses from the cohort due to mortalities and progression to disease.

The dynamics of the transmission process and specifically the probability of TB transmission in a person-to-person encounter need to be analyzed. This means that the relationship between the degree of infectiousness of an infectious person (active case) and the number of susceptible people likely to be infected by that particular infectious individual needs to be considered. The degree of infectiousness and the number of susceptible people are both functions of time.

The total number of infectious individuals in the community must also be taken into account in order to determine whether or not the cohort of infected persons is increasing in number.

### The relationship between time and degree of infectiousness

At the onset of disease, the number of bacteria exhaled by an actively diseased person will be low. In addition, we assume that coughing, the most important mechanism for the excretion of bacterium-laden droplets, will be relatively infrequent. Therefore, the risk that a person in the vicinity of an actively diseased person will breathe in bacteria and become infected is minimal. As time goes by and the disease becomes progressively more acute, the number of bacteria exhaled per unit of time increases to the point where the diseased person becomes significantly infectious. This increase will continue and eventually reach a ceiling, depending on the individual. These concepts have been formalized by a ‘risk index’ which is defined as the product of the score of the sputum-smear examination (Gaffky number) by the duration of symptomatic period in months [6]. It was concluded that the risk index was useful to predict the infectiousness of a tuberculosis patient [6]. It should be noted that models that have been developed until now assume a constant degree of infectiousness. This assumes or implies that a diseased person is either infectious and exhales the same quantity of bacteria each day until he receives treatment or he is not infectious at all. This simplifying assumption does not necessarily invalidate a model where it is applied, but is, however, not appropriate in this particular study which focuses on the issue of the degree of infectiousness itself.

Transmission is unlikely in casual, non-repeated short encounters and can therefore be ignored as being relatively insignificant. Transmission is far more likely in situations involving exposure for extended periods, or repeatedly, particularly in poorly ventilated spaces which would typically occur in households, certain workplaces and public places such as transport vehicles, churches, clubs and bars [7]. The concept of a personal contact cohort (PCC) is proposed and defined as a group of individuals who share daily and prolonged contacts (e.g. people sharing a household, workplace, or a common locality intensely) with a particular person, this person being termed the primary member. Thus each person, α, has his own PCC, denoted by PCC (α), of which he is the primary member. An epidemiologically active PCC (henceforth EPCC) is a PCC where the primary member has become infectious. Note that any of the members of a PCC may have been infected at some time in their recent or distant past and any such person may develop active disease at any time. As the prime member of his own PCC he will then have converted his PCC into an EPCC. During the time while he is infectious it is extremely unlikely that any other member of his PCC will also become infectious as the time scales involved are totally different.

**Figure 1:**
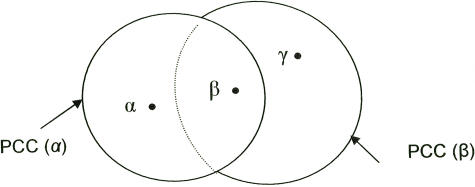
Different individuals belonging to a particular PCC each have their own non-coinciding PCC's.

These definitions differ from those for generalized households or clusters and epidemiologically active generalized households or clusters [7], [8]. A generalized household or cluster is a collection of people who regularly have extended close contact, such as members of a family living in one dwelling or people sharing a workplace. The definition assumes that any one of the members is equally likely to infect any other member and unlikely to infect anyone outside the cluster. Thus all members of all the families of people sharing a workplace are automatically regarded as part of a cluster. The definition of a PCC is far more exclusive: the important difference is that although two PCC's may have members in common they are not automatically identical cohorts. So although PCC (α) and PCC (β) may share members, not every member of PCC (β) is susceptible to infection by α. This will still be the case even if β is a member of PCC (α) (figure 1). This figure shows that if the prime member α becomes infectious then the member β of PCC (α) and PCC (β) will become vulnerable to infection by α. However γ will not be vulnerable to infection directly by α since γ does not belong to PCC (α).

A PCC will gain new members as children become old enough to be potential pulmonary TB cases. The same PCC will be losing members through mortality at an equal rate since, as stated in the introduction, the birth and mortality rates are assumed equal for the community under consideration. Thus the flux of people through the PCC will maintain the age structure of the PCC and will not in itself cause a change in the relative proportions of infected and susceptible individuals. In fact, the rates of recruitment and mortality and even migration, if that is to be considered, are insignificant compared to the rate of infection that occurs in an EPCC. Typically an EPCC will produce about six to ten new instances of infection in a matter of a few weeks. During this short time period, the number of mortalities, recruitments and migration will most likely be nil, so a stable population size is not a requirement. Nevertheless the simplifying assumptions are retained in order to keep the presentation of the equations as clear as possible.

**Figure 2:**
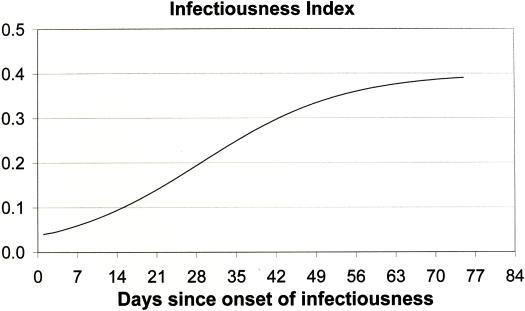
The infectiousness index function, *f,* relating degree of infectiousness at time *t* days since the onset of disease in the primary member of the PCC.

For a person with active disease and one other susceptible member of his or her PCC, the probability of effective transmission of TB during a contact event between them depends on the extent of the infectiousness of the person with active disease. The level of infectiousness is a function of the time since the person with active disease became infectious and is called the *infectiousness index*. The infectiousness index is low for a person who has only recently become infectious and increases as the person becomes more ill [6]. This increase may be assumed to be approximated by an exponential growth curve as this is the manner in which the bacteria infecting a person probably multiply. The increase will eventually tend to a saturation value. So the infectiousness index can be approximated by a logistic function, *f*, as shown in figure 2 (see Appendix S1 for details). The risk of an infection event occurring will depend on the level of susceptibility of the individual exposed to an infectious person. By assuming a mean value for the degree of susceptibility, the infectiousness index will have values that are independent of the particular person being exposed to a potential infection event.

### The number of available susceptible contacts

A PCC becomes epidemiologically important when the primary member develops active TB disease. Such an individual most likely has a limited number of potential close contacts so the size of the PCC may be relatively small. In industrialized countries the median size is about six with reports of up to more than ten [10]–[12]. But the number may well be considerably larger, particularly where extensive and densely occupied areas are involved. Under such conditions, in an EPCC, as many as 40 or more direct infection events have been reported [13]. However, such large numbers of direct infections occur under unusual circumstances and should not be regarded as typical.

The infectious primary individual will, by definition of a PCC, encounter most of these contacts within the initial weeks after onset of disease. During these weeks while the infectiousness of the primary member is still low, the rate at which members of his PCC become infected is relatively slow. Initially therefore, the number of potential susceptible contacts decreases slowly. As time progresses, the primary member becomes increasingly infectious and the number of remaining uninfected contacts decreases more rapidly. Eventually, the remaining members of the PCC will typically be those uninfected people in the PCC that have less intense contact with the primary person. It will take longer for such people to experience sufficient contact with the primary person to enable infection. Moreover, the infectious person is likely to have become less mobile and to meet with fewer of the members of his PCC. Thus the rate of decrease in the number of uninfected susceptible people in the PCC eventually starts to diminish. The number of uninfected members of an EPCC therefore decreases over time according to a logistic curve, *g* (figure 2). Details of the derivation of this function are provided in Appendix S1.

A person who is not a member of an EPCC may be infected by the infectious primary member of that EPCC as the result of a pure chance encounter. This eventuality is referred to as infection by casual contact. By definition of the concept of a PCC, such an event would be exceptional as it would require intensive exposure by the prime member to persons that are not members of his or her PCC. Moreover, as the prime member becomes increasingly ill, he or she will become less mobile and casual contacts less likely.

**Figure 3:**
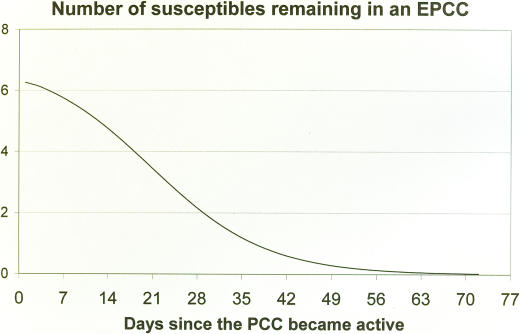
The function, *g,* describing the number of susceptible people remaining in a PCC at time *t* days since the onset of disease in the primary member of the PCC.

The focus here is on a setting typical of underdeveloped countries where high density living and work conditions prevail. In such conditions most people will have regular daily work and leisure routines and will encounter the same group of people each day. Travel far from home which would imply contact with strangers can be practically discounted. In these settings the incidence of infection events involving casual contacts can be regarded as insignificant. The functions *f* and *g* in figures 2 and 3 govern the rate at which people are infected, at time *t*, by the primary member of an EPCC as discussed in the following section.

### The total number of infected individuals in the community

A person who becomes infected may progress to active disease within a short time (a matter of a few weeks) or at any time during the course of his or her life or even not at all. The risk of progression to disease varies also according to many factors, such as age at which infection occurs, current age of the individual, gender and immunocompetency. An average value for the rate at which infected individuals in the community become infectious can be used, as this approach is consistent with the way the functions *f* and *g,* described in the previous sections, are constructed. We denote this rate by *r*. Then, at a given time *t*, the rate σ at which infected individuals in the community as a whole convert to the infectious state is given by σ = *r*·*I*, where *I*, is the total number of infected individuals at that time. This means that the rate at which newly infectious individuals appear from within the community is σ, assuming, as discussed earlier, that there is no net migration of infectious persons into or from the community under study. If, as it is reasonable to suppose, this rate σ is approximately constant over a moderate period of time such as two or three months, the total number of new infectious individuals in the community is given by σ·τ where τ is the mean time from onset of infectiousness to diagnosis and effective treatment and σ is the mean value of the conversion rate in the community as a whole over the period τ. A unique EPPC is associated with each infectious individual and the current number of EPCC's is σ·τ.

**Figure 4:**
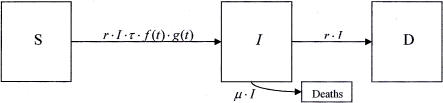
Flow diagram for the cohort of infected people.

The functions *f* and *g* are defined in such a way that the rate at which susceptible people in an EPCC become infected may be expressed as *f* (*t*), the degree of infectiousness of the primary member, multiplied by the number of potential susceptible contacts, *g*(*t*), Here *t* denotes the time from when the primary member became infectious. The rate at which people in an EPCC are being infected is given by *f* (*t*)·*g* (*t*). Since the number of EPCC's is σ·τ, it follows that in the community as a whole, the rate at which individuals become infected can be expressed as:
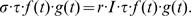
The equation governing the rate of change of *I* is constructed by considering the rates of flow into and out of the cohort of infected persons. Figure 4 is the flow diagram. It uses the terminology: S, *I* and D are the numbers of susceptible, infected and infectious diseased people, respectively in the community. *r* is the rate of progression from the infected state to the diseased and infectious state and µ is the weighted mortality rate for infected people. Note that this diagram only shows flows into and out of the compartment of infected people. These flows already encapsulate the flows into and out of the compartments for susceptible and diseased people and these flows are therefore not shown here. From figure 4 it can be seen that this rate of change can be formulated as follows:
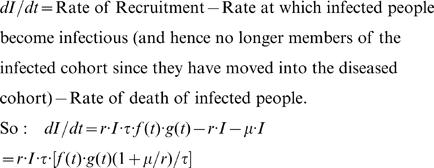
where μ is the weighted death rate of infected individuals and uninfected individuals in a PCC.

Note that the terms *dI*/*dt* and *f* (*t*)·*g* (*t*) are only loosely related although a high value of the latter compared to (1+μ/*r*)/τ would imply a positive value for *dI*/*dt*. A relatively low value for *f* (*t*)·*g* (*t*) would result in a negative value for *dI*/*dt*.

The key factor in the above equation is the term: *f* (*t*)·*g* (*t*)−(1+μ/*r*)/τ. Denote the expression (1+μ/*r*)/τ that occurs in this term by κ. To achieve control of a TB epidemic, then, at no time *t* prior to diagnosis should the value of *f* (*t*)·*g*(*t*) exceed κ. It is appropriate to refer to κ as the ceiling value for *f* (*t*)·*g*(*t*).

When an epidemic is expanding, the number of infected people is increasing. This means that the average value of *dI*/*dt* is positive for the EPCC's. Thus the situation will be that *dI*/*dt* exceeds the term κ (Kappa) for some minimal value of *t*. This minimal *t* value is called the threshold value and is denoted by Γ (Gamma).

**Figure 5:**
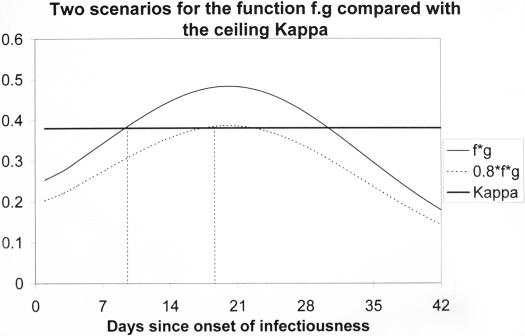
The horizontal line labeled Kappa (κ = 0.38) indicates the ceiling value for *f* (*t*)·*g* (*t*).

Gamma is the time *t* where Kappa intersects the graph of *dI*/*dt* (figure 5). Recall that the time to diagnosis is denoted by τ and the total number of infected people continues to increase while *dI*/*dt*>0. To reverse this situation, any contemplated control measures must ensure that *f* (*t*)·*g* (*t*) does not exceed κ for any value of *t* less than τ. This means that the time to diagnosis, τ, has to be less than the threshold time Γ.

## Results

The value of κ was estimated using data from a community situated in the Western Cape, South Africa. High incidence rates of TB have been endemic here for several decades. During most of this time the incidence of HIV has been negligible [9] so the complications involved in accounting for the effect of HIV on the TB epidemic do not apply to this study. The functions *f* (*t*) and *g* (*t*) and hence *f* (*t*)·*g* (*t*) were constructed using data from the same community (see Appendix S1). These functions and κ will vary from setting to setting and they will probably change slowly over time. The results presented in this section are provisional and are based on an analysis of unpublished clinical data specific for the community alluded to above. Further investigations are needed to determine definitive parameter values for the construction of these functions *f* and *g*. Nevertheless, since the incidence of TB has been increasing in this community, a situation corresponding to scenario 1 as depicted in Figure 5 must prevail. The precise extent by which the maximum value of *f* (*t*)·*g* (*t*) exceeds the value of κ is, however, not known at this stage. Figure 5 shows the function *f* (*t*)·*g* (*t*) compared to the value of κ for two scenarios. The value of κ is 0.38 (see Appendix S1). Scenario 1 corresponds to the existing situation and scenario 2 represents a situation in which *f* (*t*)·*g* (*t*) has been reduced by 20%. In scenario 1 the ceiling κ is exceeded already by day 9 whereas in scenario 2 κ is exceeded only by day 18 so that the window of opportunity to achieve a diagnosis, that is, the threshold time Γ, has been doubled. A slight further reduction in the value of *f* (*t*)·*g* (*t*) to prevent the ceiling ever being exceeded would be sufficient to bring the epidemic under control no matter how long the prevailing mean delay time to diagnosis. Alternatively, the same results could follow if measures were put in place so that a larger value of κ emerged as this could allow for an increase in the threshold time Γ to a value exceeding the mean delay time to diagnosis.

## Discussion

Three pivotal parameters have been identified that have importance for the reduction in the incidence of TB: The mean time τ, from onset of active disease to diagnosis, a threshold time Γ and a ceiling time κ. Recall that κ denotes (1+μ/*r*)/τ where μ is the mortality rate and *r* is the rate of progression from infection to disease. Any attempt to control a TB epidemic has to address the issues associated with these parameters. Control measures should intervene in such a way that *f* (*t*)·*g* (*t*) remains less than κ for all values of *t* up to the mean time to diagnosis τ.

Figure 5, taken together with the definition of κ, shows that the mean time to diagnosis, τ, is critical. A reduction in τ has an unexpected and beneficial dual effect in that this leads not only to a situation where *f* (*t*)·*g* (*t*) does not exceed κ for *t* less than τ, but also the value of κ is simultaneously increased. This results from the presence of τ in the denominator of (1+μ/*r*)/τ and assists in achieving the goal of preventing *f* (*t*)·*g* (*t*) exceeding the ceiling.

**Figure 6:**
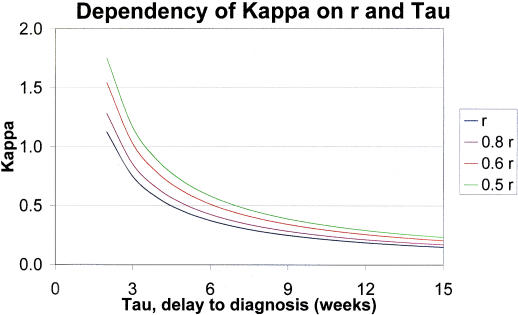
κ = (1+μ*/r*)/τ is a function of *r* and tau (τ), κ is plotted for values of *r* given by *r*, 0.8 *r*, 0.6 *r* and 0.5 *r.*

As explained later, the value of κ itself can be directly increased. If this achieved then a concomitant increase in Γ occurs, as can be seen in figure 6.

Thus to bring a TB epidemic under control one or both of two objectives need to be addressed. These objectives are the reduction in the mean delay time to diagnosis (and commencement of treatment) and a reduction in the rate of progress from infection to active infectious disease. Note that the latter objective is equivalent to reducing the number of people who progress from infection to active infectious disease. As detailed below, a variety of measures can be introduced to achieve these objectives.

Firstly, the time to diagnosis is unnecessarily extended by the logistics of acquiring, testing and reporting on a sputum sample obtained from a suspected case who has reported to a clinic. There are many potential delays before the result is available to the clinic and patient in order to initiate therapy. In particular, culture diagnosis is slow to provide a result. Rather, the quickest available diagnostic tools should be employed. Fortunately, the development of rapid diagnostic techniques is an area that is currently receiving urgent attention [14]–[21]. With on-the-spot diagnoses the other delays mentioned already would be eliminated and the only delay left would be the time from onset of systems to presentation at a clinic. Educating the public regarding the symptoms of TB and the importance of seeking medical attention at an early stage would help reduce this delay. Moreover, once it becomes generally known that immediate diagnoses are available, patients would probably view the process more favorably and be more inclined to seek medical attention.

These issues are not too difficult to address and it is possible that the time from onset of symptoms to commencement of therapy could be reduced to about two weeks. It would be necessary for the community to be educated concerning the symptoms of TB and to be aware of the need for prompt attention. Aggressive screening of the PCC of a confirmed TB case would reinforce this aspect. This measure also, should not be too difficult to put into practice and would also have the effect of reducing *r* since fewer infected people would progress to disease.

Secondly, there are benefits to be gained by effecting a reduction in *r*, the rate of progression to disease. Such a reduction can be achieved by addressing issues such as:

1. Nutrition
2. Vaccination
3. Screening
4. Moderation of lifestyle risk factors such as substance abuse, e.g. smoking and alcohol consumption
5. General health care, including nematode control and provision of sanitation and safe water supplies
6. Accommodation and working conditions.

One may expect the effects of improvement in these environmental factors to be cumulative. These measures all have the general effect of improving the general health and hence innate immunity of the population and mirror the improving living conditions noted in Europe from the 1830s onwards. During this period, as has been comprehensively documented [22], the prevalence of TB dropped dramatically from very high levels, prior to the advent of antibiotic therapy.

The concepts in this paper have been illustrated using data from a low-income community. The people frequently live in over-crowded poorly ventilated accommodation, are under-nourished, considerable substance abuse occurs and unemployment is high. These adverse conditions all need to be reversed but this will clearly take time. Only then will the value of *r* for this community be reduced. Improvement in any of these areas may also reduce the number of susceptible people, that is, reduce the size of the typical PCC, and hence decrease *f* (*t*)·*g*(*t*). This is precisely one of the goals that should be worked towards, as described earlier.

The question of drug resistant TB has not been addressed in this paper. The incidence of resistant TB is extremely low (about eight cases per annum [9]) in the community under study and the kind of analysis used here to examine susceptible TB is unsuitable for such small numbers because the concept of mean value for incidence rate, for instance, is undermined by random fluctuations. Moreover there is a special difficulty specific to drug-resistant TB; typically it is realized that a patient in therapy has a resistant TB strain only after an extended period of time. In this respect it seems highly desirable that at the time of initial diagnosis it should be established whether or not the patient will be susceptible to treatment using first-line drugs.

It is encouraging to observe that measures to control an epidemic, as discussed above, could actually have results out of proportion to that expected on an intuitive basis. This is because of the dual effect resulting from a reduction in the delay to diagnosis or a decrease in the rate of progression from infection to active infectious disease, whereby an improvement in the one induces an improvement in the other. Conversely, neglect in any area aggravates an epidemic in insidious ways and should not be tolerated in a community that is in crisis.

In summary, then, attention has been focused on the two strategies available for achieving control of a TB epidemic. The first is to reduce the value of *f* (*t*)·*g* (*t*) and the second is to increase the value of the ceiling κ. Each of these strategies increases the threshold time Γ and provided this increase is sufficient for Γ to exceed the mean delay time to diagnosis, control of a TB epidemic is feasible. A combination of both strategies would probably be the most readily achievable and cost effective approach. From the point of view of policy makers decisions have to be made on the basis of the most cost effective allocation of resources that ameliorate the various factors discussed here that contribute to the perpetuation of a TB epidemic. There is a clear need for a multifactorial economic analysis that covers the entire gamut of issues. Quite apart from the socio-economic changes needed over a long period, faster diagnosis, active case-finding, contact investigation and case follow-up is clearly required. A reactive approach of simply treating patients as they present is inadequate to deal with a major TB epidemic and may well be far more expensive in the long term than a proactive approach such as that advocated here.

## Supporting Information

Appendix S1 Appendix
(0.06 MB DOC)
