# Supplementary material for: A Threshold Value for the Time Delay to TB Diagnosis
Source: PLoS One. 2007 Aug 22;2(8):e757. doi: 10.1371/journal.pone.0000757 (PMC1942086; doi:10.1371/journal.pone.0000757)
Supplement: Appendix S1 — Appendix (0.06 MB DOC) [file pone.0000757.s001.doc]

**Appendix S1**

**The value of** *κ*

Recall that *κ* denotes (1 + *μ / r* ) / *τ* and that this expression defines a function of *τ*. For the community under study the mortality rate is 1.5% and the rate of progression to disease is 1.2%. This latter figure is calculated from the annual incidence of (new pulmonary) disease at 240 with about 20,000 people infected (60% of the population). The graphs in Figure 6 were derived using these values and a range of values for *r*.

**The logistic function *f***

Methods for measuring the degree of infectiousness of TB patients have been developed [1]. But direct measurement of the degree of infectiousness over time of a person not yet receiving therapy is obviously not possible except in an inadvertent way: For patients receiving treatment but infected with drug-resistant TB measurements of infectiousness taken over a period of time would reveal failure of response to the treatment, and would provide a way of estimating the rate of increase of infectiousness over time. The ‘risk index’ [2] indicates that until effective treatment commences, the degree of infectiousness will increase. The assumption is made that the increase will eventually slow down. A reasonable approximation to the manner of the increase is thus given by a logistic function.

A standard form for the logistic function is *f* ( *t* ) = [*C.*α*.*exp(β *t*)] / [1 + α*.*exp(β *t*)]. For large *t* this will approach the value *C* whereas for small *t* the function value will approximate *C.*α */* (1 *+* α). The value of α is selected so that *f* (0) has a relatively small value of about 0.05. A suitable value for α is 0.1. Typically, a primary member of a PCC will infect about half of the members of the PCC but even more members may be infected in some circumstances [3–6]. We have taken a conservative view and assumed that 40% of the members of an EPCC become infected. This corresponds to setting the ceiling value for the function *f* at 0.4. So this is the value assigned to *C*. The parameter β measures the exponential rate of growth of the infection and its value was selected to yield a time to 50% of maximum infectiousness of 35 days. So, in other words, the ‘half-life’ was set at 35 days. It is not possible to determine a specific value for β at present but the effects of several values of β in the range 0.125 to 0.0125 can be investigated. This corresponds to a time of progression from onset of disease to a stage of severe infectiousness in the range 40 to 70 days.

The value of *κ* is calculated using available data, and has the value of 0.38 given that the mean delay to diagnosis in this community is believed to be about six weeks. Figure 5 indicates that, since under existing conditions of endemic TB, the value of *f* (*t*)*. g*(*t*) must exceed *κ* before diagnoses are being achieved; *f* (*t*)*. g*(*t*) achieves a value of at least 0.38 at some time *t*, before the sixth week.

The function *g* is given by N – *h* where N is the average number of people in a PCC and *h* is another logistic function constructed using the principles as were used for the function *f*. Once again, the value for α is set at 0.1. The value of *C* is an estimate of the average PCC size. The rate at which PCC members become infected will increase as the primary member becomes more infectious. So the ‘half-lives’ of the *f* and the *g* functions will be positively correlated. This in turn implies that the values of β for these two functions will be similar. Again a variety of values of β for the *g* function need to be investigated.

Parameter values used in the construction of these functions as shown in figures 2 and 3 are provided in Table 1.

**Table 1: Parameter values**

| **Function**  *f* | **Value** | **Function**  *g* | **Value** |
| --- | --- | --- | --- |
| *C* | 0.5 | *C* | 16 |
| α | 0.1 | α | 0.1 |
| β | 0.125 | β | 0.125 |
| Estimated half-life | 35 days | Estimated half-life | 30 days |

**Note: Parameter values are estimates based on data for the community under consideration**

**References**

1. Fennelly KP, Martyny JW, Fulton KE, Orme IM, Cave DM et al. (2004) Cough-generated aerosols of *Mycobacterium tuberculosis*: a new method to study infectiousness. Am J Respir Crit Care Med 169: 604-609.

2. Toyota M (1994) Validity of "the risk index" to predict the infectiousness of tuberculosis patients. Kekkaku 69: 375-377.

3. Reichler MR, Reves R, Bur S, Thompson V, Mangura BT et al. (2002) Evaluation of investigations conducted to detect and prevent transmission of tuberculosis. JAMA 287: 991-995.

4. Marks SM, Taylor Z, Qualls NL, Shrestha-Kuwahara RJ, Wilce MA et al. (2000) Outcomes of contact investigations of infectious tuberculosis patients. Am J Respir Crit Care Med 162: 2033-2038.

5. Bailey WC, Gerald LB, Kimerling ME, Redden D, Brook N et al. (2002) Predictive model to identify positive tuberculosis skin test results during contact investigations. JAMA 287: 996-1002.

6. Phillips L, Carlile J, Smith D (2004) Epidemiology of a tuberculosis outbreak in a rural Missouri high school. Pediatrics 113: e514-e519.
